# Missing HIV prevention opportunities in South African children – A 7-year review

**DOI:** 10.1186/1471-2458-14-1265

**Published:** 2014-12-13

**Authors:** Ute D Feucht, Anell Meyer, Mariana Kruger

**Affiliations:** Department of Paediatrics, University of Pretoria, Pretoria, South Africa; Department of Paediatrics and Child Health, University of Stellenbosch, Tygerberg, South Africa

**Keywords:** HIV, Prevention of mother-to-child transmission (PMTCT), Missed opportunities

## Abstract

**Background:**

The prevention of mother-to-child transmission (PMTCT) program in South Africa is now successful in ensuring HIV-free survival for most HIV-exposed children, but gaps in PMTCT coverage remain. The study objective was to identify missed opportunities for prevention of mother-to-child transmission of HIV using the four PMTCT stages outlined in National Guidelines.

**Methods:**

This descriptive study enrolled HIV-exposed children who were below the age of 7 years and therefore born during the South African PMTCT era. The study site was in Gauteng, South Africa and enrolment was from June 2009 to May 2010. The clinical history was obtained through a structured caregiver interview and review of medical records and included socio-demographic data, medical history, HIV interventions, infant feeding information and HIV results. The study group was divided into the “single dose nevirapine” (“sdNVP”) and “dual-therapy” (nevirapine & zidovudine) groups due to PMTCT program change in February 2008, with subsequent comparison between the groups regarding PMTCT steps during the preconception stage, antenatal care, labor and delivery and postpartum care.

**Results:**

Two-hundred-and-one HIV-exposed children were enrolled: 137 (68%) children were HIV infected and 64 (32%) were HIV uninfected. All children were born between 2002 and 2009, with 78 (39%) in the “sdNVP” and 123 (61%) in the “dual-therapy” groups. The results demonstrate significant improvements in antenatal HIV testing and PMTCT enrolment, known maternal HIV diagnosis at delivery, mother-infant antiretroviral interventions, infant HIV-diagnosis and cotrimoxazole prophylaxis. Missed opportunities without improvement include pre-conceptual HIV-services and family planning, tuberculosis screening, HIV disclosure, psychosocial support and postnatal care. Not receiving consistent infant feeding messaging was the only PMTCT component that worsened over time.

**Conclusions:**

Multiple missed opportunities for optimal PMTCT were identified, which collectively increase children’s risk of HIV acquisition. Although HIV-testing and antiretroviral interventions improved, all PMTCT components need to be optimized to reach the goal of total pediatric HIV elimination.

**Electronic supplementary material:**

The online version of this article (doi:10.1186/1471-2458-14-1265) contains supplementary material, which is available to authorized users.

## Background

The prevention of HIV transmission to children has become an achievable goal, even in high-prevalence countries like South Africa, where almost one third of all children are born to HIV-infected mothers. This prospect of pediatric HIV elimination is attributable to continued advances in the prevention of mother-to-child transmission (PMTCT) program, resulting in reduced numbers of infant HIV infections and improved outcomes for mother-infant pairs [1].

The South African PMTCT program was implemented in 2002 [2, 3], initially using single-dose nevirapine (“sdNVP”) at the time of delivery for both mother and baby, with the addition in April 2004 of antiretroviral therapy (ART) for women with severe HIV disease, as part of the countrywide ART rollout. Severe disease was classified as World Health Organization (WHO) stage 4 disease or a CD4 count ≤200 cells/μl [4]. The PMTCT program was revised in 2008 [5], when the antiretroviral strategy changed to “dual-therapy”, with the addition of antenatal (third trimester) and postnatal (infant) zidovudine (AZT). Although ART initiation criteria remained unchanged, more focus was placed on expanding access to maternal triple therapy to ensure health and survival. Additionally four PMTCT stages were defined: preconception (stage 1), antenatal period (stage 2), labor and delivery (stage 3) and postnatal period (stage 4); the last-mentioned of these four including the early identification of HIV-infected children to ensure optimal survival. PMTCT program revision again occurred in 2010, extending antenatal zidovudine to the second trimester, elevating the maternal CD4 threshold for ART eligibility to 350 cells/μl and including WHO stage 3 disease, and the addition of ART prophylaxis during breastfeeding [6]. Since 2013 South Africa has moved to triple-ART interventions during pregnancy and breastfeeding for all mothers. There is also a movement towards improved integration of PMTCT interventions into routine maternal- and child-health services [7].

PMTCT interventions are highly effective, with HIV-transmission rates of <5% achievable even in breastfeeding populations [8]. PMTCT being a multistep program may have its disadvantages, though, as quality assurance and continuity of care are necessary along the entire pathway [9–11]. The study aim was to investigate potential missed opportunities in PMTCT, as well as to compare the intervention coverage in the early sdNVP era with the subsequent dual-therapy period. The identification of potential problematic areas may assist in improving PMTCT implementation to ensure further reduction in HIV transmissions with improved outcomes for affected mothers-infant pairs.

## Methods

This descriptive study was conducted between June 2009 and May 2010 at Kalafong Hospital in Gauteng, South Africa. HIV-infected- and HIV-exposed-but-uninfected children below the age of 7 years, who were in care at the hospital’s pediatric services, were recruited, as they had been born during the South African PMTCT era. A detailed clinical history was obtained by conducting a structured interview with the primary caregiver using a questionnaire designed to describe the mother-infant’s path through the four PMTCT stages, with emphasis on missed opportunities (preconception advice, maternal HIV diagnosis, medical interventions, psychosocial support, feeding counseling and infant follow-up). See Additional file 1. Additional relevant information was obtained from the child’s medical records, including hospital records and the patient-held Road-to-Health Card, in addition to maternal medical records if these were available. Data collected from the health records included socio-demographic data, medical history of the mother and child, HIV interventions and infant feeding information. Furthermore we reviewed the National Health Laboratory Service database for the HIV results of study participants.

The study group was divided into two groups, based on the PMTCT program at the time: The “sdNVP” group, born before February 2008, and the “dual-therapy” group born thereafter. Missed opportunities were identified and grouped together for descriptive purposes. Antenatal ART and postnatal ART prophylaxis had not been part of PMTCT from 2002 to 2007 and were therefore not included as missed opportunities in the comparison of the two cohorts.

The Ethics Review Committee, Faculty of Health Sciences, University of Pretoria, approved the study protocol. Descriptive statistics were used to describe participants’ characteristics and missed PMTCT opportunities. Statistical analysis was performed using SPSS® version 20. Categorical variables were examined using two-sided chi-square or Fisher’s exact tests, with p-values ≤0.05 considered significant.

## Results

Two-hundred-and-one HIV-exposed children were enrolled: 137 children (68%) were HIV infected and 64 (32%) were HIV uninfected, and all born between 2002 and 2009. There were 75 (37%) in the “sdNVP” group and 126 (63%) in the “dual-therapy” group. For socio-demographic and medical characteristics of the study population see Table 1.

**Table 1:** **Socio-demographic and medical characteristics of the study population (n = 201)**

**Housing information**	• Permanent brick structure	119 (59%)
	• Running water indoors	93 (46%)
	• Electricity/gas/paraffin available	153 (76%)
**Main source of household income**	• Regular income employment	92 (46%)
	• Temporary employment	30 (15%)
	• Social grants/grandparent’s pension	79 (39%)
**Mode of transport used for travel to health services**	• Public transport/communal taxi	191 (95%)
	• Private transport	9 (4.5%)
	• Travel on foot	1 (0.5 %)
**Child’s rank within family**	• 1st child	71 (35%)
	• 2nd child	72 (36%)
	• 3rd child or more	58 (29%)
**Antenatal care**	• None	8 (4%)
	• One to three visits	66 (33%)
	• Four to six visits	101 (50%)
	• Seven visits and more	26 (13%)
Maternal HIV test*	• Before pregnancy	32 (16%)
	• First trimester	33 (16.5%)
	• Second trimester	102 (51%)
	• Third trimester	10 (5%)
	• After pregnancy	23 (11.5%)
**Paternal HIV status**	• Positive	52 (26%)
	• Negative	4 (2%)
	• Never tested/unknown	145 (72%)
**Place of delivery**	• Health care facility	192 (96%)
	• Home	9 (4%)
**Type of delivery**	• Normal vaginal delivery	142 (71%)
	• Cesarean section	59 (29%)
Birth weight**	• Less than 2.5 kg	23 (11%)
	• 2.5 - 4 kg	172 (86%)
	• More than 4 kg	5 (3%)
**Infant feeding choice at the time of delivery**	• Exclusive breastfeeding	135 (67%)
	• Formula feeding	66 (33%)

*One case excluded as timing of maternal HIV test unknown.

**One case excluded as birth weight unknown.

At study enrolment, 13 mothers (6%) had died, and 16% of children were cared for by someone other than their mother. The maternal CD4 count was known in 58%, with 17% on ART, while 16% had been treated for tuberculosis (TB). Almost half of caregivers (48%) reported previous treatment by traditional healers. The mean maternal age at the child’s birth had been 27 years (range 16–46 years), and 32 years (range 18–64 years) for fathers. Twenty-four fathers (12%) had died, two were clinically unwell, and in 8% of cases the father’s whereabouts was unknown. Only 52 fathers (26%) were known to be HIV positive, with 10% on ART. Caregivers knew the father’s CD4 count in 7 cases (3%) and 6% reported paternal TB treatment. One-hundred-and-thirty-eight study participants (69%) had siblings, but in 61% of these families none of the siblings had been tested for HIV. Five siblings were HIV positive, two of them receiving ART, while 6 siblings were reported unwell. Another 7 siblings had died, of which 6 deaths had been HIV-related, while the seventh child had died in a motor vehicle accident.

### Missed opportunities

**Figure 1:**
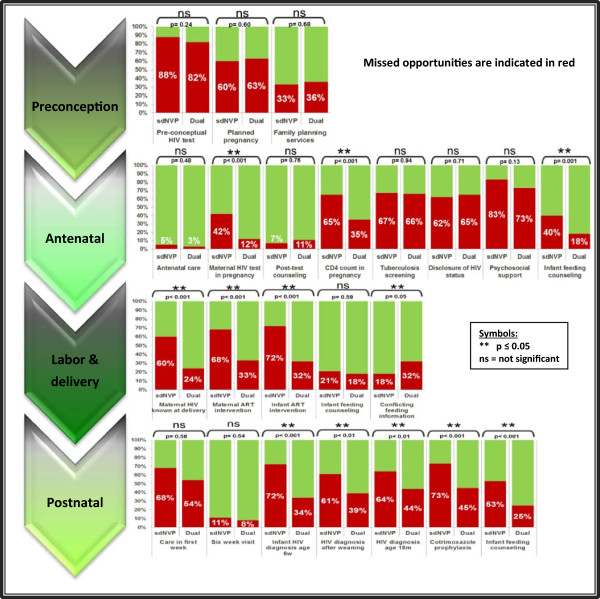
**Comparison of the missed PMTCT opportunities in the “sdNVP”-group and the later “dual-therapy” group.**

Missed opportunities were identified in all four PMTCT stages and are shown in Figure 1 and discussed comparing the “sdNVP” and “dual-therapy” groups.

### Preconception care

Pre-conceptual PMTCT components include identification and education of HIV-infected women of childbearing age regarding strategies for prevention of unplanned pregnancies and HIV transmission. The HIV status of 88% of “sdNVP-” and 82% of “dual-therapy” mothers had been unknown prior to conception, which was comparable between the groups (p = 0.24; NS). Thirty-two mothers had known their HIV status before pregnancy, including seven who had had no pre-conceptual counseling regarding the possibility of HIV transmission, nor regarding risk-reduction strategies. No significant improvements existed in the other two pre-conceptual care activities assessed as, in total, 123 of the pregnancies (62%) had been unplanned or unwanted (p = 0.60; NS), while mothers reported barriers to family planning services in 35% of cases (“sdNVP” = 33% (25/75); “dual-therapy” = 36% (45/126); p = 0.68, NS).

### Antenatal care

The second PMTCT stage focuses on identification of HIV-infected pregnant women and the provision of appropriate antenatal healthcare. Antenatal care attendance had been high, with both groups having had only four mothers not in care (p = 0.48, NS). Almost two-thirds (63%) had had at least four antenatal visits, with ample opportunity for PMTCT enrolment. The majority of maternal HIV tests had been done in the second trimester (51%). Mothers for whom no antenatal HIV test had been done decreased significantly from 42% in the “sdNVP” group to 12% in the “dual-therapy” group (p < 0.001). Fifteen mothers had had inadequate post-test counseling, with no significant differences between the two groups (p = 0.76, NS).

Antenatal CD4 counts had not been taken in 65% of HIV-positive mothers in the “sdNVP” group – a missed opportunity – but this percentage significantly improved to 35% in the “dual-therapy” group (p < 0.001). The median waiting time for CD4 results had been 4 weeks. Fifteen mothers (7%) reported that no information had been given regarding the importance of the CD4 count, while seven mothers had never received their CD4 results. Two mothers from the “sdNVP” group had started ART during pregnancy, with one already on ART prior to conception. In the “dual-therapy” group, 36% of mothers had received AZT from 28 weeks, while 2% had started on ART and another 2% had never returned for treatment initiation. Screening for TB had not been carried out in 67% and 66% of mothers in the respective groups (p = 0.94, NS).

Non-disclosure of their HIV status was reported by 62% of “sdNVP” mothers, comparable to 65% in the subsequent period (p = 0.71, NS), while psychosocial support had not been offered to 83% and 73% respectively (p = 0.13, NS). Mothers reported no antenatal infant feeding counseling in 40% of the “sdNVP” group, with significant improvement to 18% in the “dual-therapy” group (p = 0.001).

### Labor and delivery

The aim of the third PMTCT stage is to minimize the risk of HIV transmission during labor and delivery. The majority of births (96%) had occurred within healthcare facilities, but in 40% the health workers attending to the delivery had been unaware of the maternal HIV infection, a missed opportunity that significantly improved from 60% (45/75 “sdNVP”) to 24% (30/126 “dual-therapy”) (p < 0.001). ART at time of delivery had not been administered to 68% of the “sdNVP” mothers but this improved significantly to 33% in the “dual-therapy” group (p < 0.001), while the percentage of infants without appropriate antiretroviral medication also meaningfully decreased from 72% to 32% (p < 0.001).

Maternal report of receiving inconsistent infant feeding information from different health workers during and after pregnancy was the only missed opportunity that had significantly increased over time, from 18% (“sdNVP”) to 32% (“dual-therapy”) (p = 0.05). At birth, two-thirds of mothers had chosen to exclusively breastfeed their infants, with infant feeding counseling described as inadequate in 21% (16/75) and 18% (23/126) respectively (p = 0.59, NS). Twenty-five mothers (19%) had experienced difficulty with breastfeeding initiation, while another 59% stated that they would have benefited from assistance in establishing exclusive breastfeeding. Counseling had been done in 70% regarding the increased risk of HIV transmission through mixed feeding. Sixty-six mothers (33%) opted for replacement feeds. Questions assessing the suitability of this choice, by considering whether it had been acceptable, feasible, affordable, sustainable and safe at the time revealed inappropriateness in 45 mothers (68%), while 11% reported no counseling prior to commencing formula feeding.

### Postnatal care

The focus of the fourth PMTCT stage is on reduction of postnatal HIV transmission and identification of HIV-infected infants. Less than half of mother-infant pairs had received healthcare in the first week after delivery, with no improvement noted over time (“sdNVP” = 68%, “dual-therapy” = 54%) (p = 0.58; NS). The majority of HIV-exposed infants had received healthcare at 6 weeks, which amounted to 89% and 92% respectively (p = 0.54, NS), while in 38% of cases a different postnatal clinic compared to the antenatal clinic had been visited. Cotrimoxazole prophylaxis had not been given to 73% of “sdNVP” infants, but this subsequently significantly improved to 45% for the “dual-therapy” group (p < 0.001). Mothers reported no or incomplete infant feeding counseling at the six-week visit in 53% (40/75; “sdNVP”), which improved significantly to 25% (32/126; “dual-therapy”) (p < 0.001).

Initial HIV DNA PCR testing for infant diagnosis had not been done in 48%, but with significant improvement over time (“sdNVP” = 72% (54/75); “dual-therapy” = 34% (43/126); p < 0.001). Nine mothers had not obtained their infant’s HIV results. In 61% of the “sdNVP” group the post-weaning HIV diagnostic test had not been done, but this significantly improved to 39% (p < 0.01); failure to do an HIV ELISA/rapid test at age 18 months significantly improved from 64% to 44% (p < 0.01). Fifty-one infants (37%) had been diagnosed HIV positive through PMTCT testing (“sdNVP” = 10, “dual-therapy” = 41), but five children had had no CD4 count, clinical staging and referral for ART done, although the HIV DNA PCR had been positive.

## Discussion

The PMTCT program represents a continuum of healthcare towards prevention of pediatric HIV infection and optimizing maternal-infant outcomes. The WHO encouraged a comprehensive PMTCT approach since 2002, including primary HIV prevention, prevention of unintended pregnancies in HIV-infected women, PMTCT, along with care provision for HIV-positive women, their infants and families [12]. Enhanced ART interventions have since evolved, shifting the goalpost to “virtual elimination of pediatric HIV” or HIV transmission rates at <5% set as the WHO target for 2015, which emphasizes the need for renewed focus on health service improvements to ensure the availability and the uptake of all PMTCT components [13–16].

In this study we were able to show significant progress over time for several PMTCT elements, as demonstrated through improved maternal HIV diagnosis, ART interventions and infant diagnosis. However, this study identified missed opportunities in all four PMTCT stages, and in some of the steps optimal PMTCT was missed in more than half of cases, collectively increasing children’s risk of HIV acquisition. Despite emphasis towards promoting the PMTCT continuum, which requires interventions to commence even before pregnancy, reports indicate disappointing progress at implementation level regarding the pre-conceptual components [14]. Primary prevention strategies need high priority, which includes targeted HIV testing of women of childbearing age. This study demonstrated that less than one in five mothers were aware of their HIV infection before pregnancy, which correlates with another South African study, where only 12% of mothers of newly diagnosed HIV-infected children were aware of their own HIV infection prior to conception [17]. Known HIV status before pregnancy was previously proven to translate into higher PMTCT uptake and early infant testing [17]. Furthermore, almost a third of our study mothers reported difficulties in accessing family planning services, while more than half reported unplanned pregnancies, indicating that these pre-conceptual PMTCT steps require urgent attention. Linking HIV- and family-planning services is feasible and effective in reducing vertical HIV transmission and has proven to be more cost effective than ART interventions. However, most PMTCT programs continue to place the main emphasis on ART provision, as previously reported [15, 18, 19].

During pregnancy, routine healthcare and PMTCT interventions can readily be linked, especially when antenatal care utilization rates are high, like in South Africa [17]. Such linking can then translate into increased antenatal HIV testing with subsequent CD4 counts, which was confirmed in our study. However, HIV testing was done in the second trimester or later in two-thirds of our study women, limiting timely PMTCT interventions and indicating the need to focus on early pregnancy bookings. Additionally the overlapping HIV and TB epidemics pose a serious threat to HIV-infected women and their offspring, but TB screening during pregnancy was not carried out in two thirds of the study group, pointing towards a further urgent need [20]. Previous research indicates increased TB rates in HIV-positive pregnant women compared to HIV-uninfected women [21], while HIV/TB co-infection during pregnancy potentiates the risk of both HIV- and TB transmission to the infants [22, 23].

To ensure optimal medical management and to meet specific client needs, individualization of patient management should be maintained as far as possible, even within large public health programs like PMTCT. Study participants reported inadequate psychosocial support, with no improvement over time, which is of concern as many pregnancies were unplanned and most participants received their HIV diagnosis during pregnancy, adding to known emotional and financial stresses associated with pregnancy and child birth [24]. High maternal depression rates have previously been described in HIV-infected pregnant women [25], while infants of mothers with mental illnesses are at risk of insufficient maternal care and poor growth [26, 27]. This indicates the need to integrate psychosocial support and mental health services into antenatal care. Non-disclosure of HIV status, reported by two-thirds of study participants, is associated with non-attendance of HIV services as well as non-adherence to maternal-infant ART regimens, as previously described in Sub-Saharan Africa [24, 28]. A previous South African study found the current PMTCT approach to have a strong focus on the pregnant woman as an individual, with very little male participation, correlating with our findings that 72% of mothers did not know their partner’s HIV status, nor was sibling HIV testing done in 61% of families, clearly indicating the need for a more family-centered approach [11, 14, 29].

Continuity of care between pregnancy and delivery services is vital to ensure that health workers providing care during the birth process are aware of maternal HIV infection and prior interventions. A previous South African report indicated this to be problematic, because of ineffective record keeping (such as use of HIV coding systems on health records) and because health workers did not enquire about HIV or mothers did not report their HIV-status [30]. We recorded significant improvement over time in terms of the maternal HIV diagnosis being known at time of delivery, with improved maternal-infant ART provision. Continued emphasis on meticulous record keeping is needed, along with social mobilization to increase the client demand for appropriate services [30]. Discontinuation of HIV care for mothers after pregnancy has also been described in sub-Saharan Africa [28, 31]. Many (59%) of our mother-infant pairs received no healthcare in the postnatal period, a time known to be associated with a high risk of maternal and neonatal mortality. The six-week infant immunization visit, though, was well attended (>90%), but continuity of care may have been compromised, as many mothers visited different clinics post-delivery compared to their antenatal clinics, with potential loss of information between different providers. Half of the study mothers also chose to visit traditional healers, which is a finding of note, as these services fall mostly outside of the national PMTCT framework (and previously described in another South African study) [32].

Infant feeding counseling, straddling the antenatal and postnatal periods, should assist mothers in making informed choices on safe feeding. Consistent infant feeding information was the only PMTCT component which significantly worsened over time in this study, which is similar to the maternal confusion about infant feeding described in another study in Soweto, South Africa [33]. This reflects on the transition period, in which expanding scientific knowledge on ART prophylaxis for breastfeeding mothers led to adjustments in clinical practice and ultimately to changes in guidelines, without coordination at implementation level. Many women (78%) in our study voiced the need for practical help with breastfeeding initiation, clearly indicating that maternal support is essential to ensure that exclusive breastfeeding is established in all mothers [34]. Mothers who opted for formula feeding could not do so safely in 68%, placing infants at risk of associated morbidity [35, 36].

Early identification of HIV-infected infants is essential to optimize childhood outcome, but this remains challenging. According to the National Guidelines HIV DNA PCR testing should be done on all HIV-exposed children at the age of 6 weeks, followed by an age-appropriate HIV-test after weaning and an HIV ELISA/rapid test at 18 months [5]. We demonstrated improvements in HIV testing at all three time points, correlating with published South African data [3], although full coverage has not been reached. HIV-infected children that are not identified during the PMTCT process may have delayed pediatric ART initiation, with resultant high HIV-related infant mortality [14, 37]. HIV-exposed children as a risk group also need additional healthcare to mitigate the many well described health risks, such as increased rates of gastrointestinal and respiratory infections [38, 39]. Among others, they should receive cotrimoxazole prophylaxis for prevention of early opportunistic infections, which did improve over time as demonstrated in this study (from 27% to 55%), but coverage remained suboptimal.

Study limitations were that information was self-reported by primary caregivers, which in 16% was not the mother. Recall bias may have been more pronounced in the earlier “sdNVP”-group, due to the longer time lapse since PMTCT participation. Verbal information was validated using hospital records, patient-held records and laboratory databases as far as possible. Language barriers during interviews were mitigated through the use of translators. Study group allocation occurred according to date of birth; however, pregnancies last nine months and updated PMTCT protocols do not change overnight at implementation date; therefore, group allocation in those born during the transition between the two defined periods may have had limitations. Furthermore this was a hospital based study, with two-thirds of study participants being HIV infected and therefore had failed PMTCT, with high probability of missed PMTCT opportunities. As this was not a population based study, the results are thus not generalizable for all children from the PMTCT program of the time, but rather missed opportunities are highlighted through this study design.

For further improvement of PMTCT services, health systems should be improved [16, 19]. Rapid implementation of a large-scale public health intervention such as PMTCT should be followed by full integration into routine health services [40]. Missed opportunities must be identified and addressed.

## Conclusions

This study has identified missed opportunities for HIV prevention among South African children using the four PMTCT stages outlined in the National Guidelines. The data has demonstrated significant improvements in antenatal PMTCT enrolment, known maternal HIV diagnosis at delivery, mother-infant ART interventions and infant HIV diagnosis, indicating PMTCT focus areas that have succeeded. A lot still needs to be done to improve preconception HIV services linked to family planning services, TB screening in pregnancy, HIV disclosure, psychosocial support, postnatal care, including infant feeding support, in order to reach the ultimate goal of pediatric HIV elimination.

## Electronic supplementary material

Additional file 1: **Questionnaire and data collection tool.** (PDF 107 KB)
