# Supplementary material for: Missing HIV prevention opportunities in South African children – A 7-year review
Source: BMC Public Health. 2014 Dec 13;14:1265. doi: 10.1186/1471-2458-14-1265 (PMC4300827; doi:10.1186/1471-2458-14-1265)
Supplement: Supplementary file 1 — Additional file 1: Questionnaire and data collection tool. (PDF 107 KB) [file 12889_2014_7450_MOESM1_ESM.pdf]

Additional File 1: Questionnaire and data collection tool

STUDY NUMBER:			DATE:		
Date of birth:		Age:	Sex:	Male	Female
Primary care giver:	Mother	Grandmother	Father	Aunt	Other
If other, specify					
Person completing questionnaire:					

Caregiver Interview

PRIMARY PREVENTION OF HIV

Did you know how to prevent yourself from getting HIV before you fell pregnant?	Yes	No	Unknown / N/A
Did you know your HIV status before you fell pregnant?	Yes	No	Unknown / N/A
If HIV status was known before pregnancy			
Where you counseled (before the pregnancy) on the risk of HIV transmission to your baby in future pregnancies?	Yes	No	Unknown / N/A
Where you counseled (before the pregnancy) on how this risk of HIV transmission to your baby could be reduced?	Yes	No	Unknown / N/A
Planned pregnancy	Yes	No	Unknown / N/A
Where you ever counseled regarding family planning?	Yes	No	Unknown / N/A

Type of contraception used:				
If applicable, reason for not using contraception	Personal choice	Religious beliefs	Unavailable at clinic	Unaware that it is available
Other reason:				

ANTENATAL

Mom attended antenatal clinic	Yes	No	Unknown / N/A
Which clinic : Number of visits:			
Mom tested for HIV	Yes	No	Unknown / N/A
Gestation: Reason:			
Did the mother receive her HIV test result?	Yes	No	Unknown / N/A
How long did the mother have to wait for the HIV test result?			
Was post-test counseling done?	Yes	No	Unknown / N/A
Any re-testing done (if HIV test was done early in pregnancy)?	Yes	No	Unknown / N/A
Did the mother disclose her HIV-status?	Yes	No	Unknown / N/A
If yes, to whom and when			
CD4 done in pregnancy?	Yes	No	Unknown / N/A
When/Date:			
Duration between HIV test and referral for ART (if applicable):			
Absolute count : HIV viral load:			
How long did the mother have to wait for her CD4 test result?			
Was the mother informed why it was important for her to come back for her CD4 result?	Yes	No	Unknown / N/A
Referred for antiretroviral therapy?	Yes	No	Unknown / N/A
Duration between HIV-test, CD4 count result and referral for ART:			
Antiretroviral therapy:	Yes	No	Unknown / N/A
Gestation: AZT:	ART:		
Duration between referral for ART and appointment at ART clinic:			
How far is ART clinic from the patient's home?			
Counseled on safe infant feeding choices?	Yes	No	Unknown / N/A
Psychosocial support offered?	Yes	No	Unknown / N/A
Referral to social worker Referral to psychologist			
Screened for tuberculosis?	Yes	No	Unknown / N/A

LABOR AND DELIVERY

Place of birth:	Home	Clinic		Hospital	Unknown
Where the health care providers (doctors, nurses) assisting in the delivery aware of the mothers HIV status?			Yes	No	Unknown / N/A
Mode of delivery:	Normal vaginal	Caesarean section in labor due to obstetrical indication	Elective caesarean section	Forceps	Vacuum delivery
Indication for assisted delivery?					
Premature rupture of membranes			Yes	No	Unknown / N/A
If yes:	Spontaneous	Induced	< 4 hours		> 4 hours

Birth weight:	Gestation:	
ART interventions at delivery:	Mother<br>No antiretroviral treatment	Infant<br>No antiretroviral treatment
	Nevirapine alone	Nevirapine alone
	Timing of NVP	AZT 7 days
	Nevirapine and AZT	AZT 28 days
	Incomplete	Incomplete
Birth complications:		
Feeding counseling: None Doctor Sister Other:		
Did the mom ever receive conflicting information?		
Feeding choices (at birth):	Exclusive breastfeeding Pasteurized breast milk	Formula

Exclusive breastfeeding			
Problems with implementation?	Yes	No	Unknown / N/A
Received help?	Yes	No	Unknown / N/A
Needed help?	Yes	No	Unknown / N/A
Family support?	Yes	No	Unknown / N/A
How long did she give exclusive breastfeeding?			
Reason for stopping EBF?			
Was the mother counseled that mixed feeding increases the risk of HIV transmission?	Yes	No	Unknown / N/A
Pasteurization:			
Correct technique	Yes	No	Unknown / N/A
Appropriate equipment	Yes	No	Unknown / N/A
Acceptability in household	Yes	No	Unknown / N/A
Formula:<br><br>AFASS criteria<br>**A**cceptable **F**easible **A**ffordable **S**ustainable **S**afe			
Mom's reason for formula feed?			
Was the mom counseled before formula feed given?	Yes	No	Unknown / N/A
Does mom know how to mix the feed correctly?	Yes	No	Unknown / N/A
Does mom know how to hygienically prepare the formula?	Yes	No	Unknown / N/A
Did mom have the necessary equipment?	Yes	No	Unknown / N/A
Was the mom referred to the clinic to collect formula milk?	Yes	No	Unknown / N/A

POSTNATAL

Did mother have postnatal care in the first postnatal week?	Yes	No	Unknown / N/A
Appointment made for 6 week follow-up visit on discharge?	Yes	No	Unknown / N/A
Where?			
Did the mother attend?	Yes	No	Unknown / N/A
At 6 week follow-up:			
HIV DNA PCR done	Yes	No	Unknown / N/A
Bactrim prophylaxis given	Yes	No	Unknown / N/A
Received immunizations	Yes	No	Unknown / N/A
Counseled regarding feeding choices	Yes	No	Unknown / N/A
Was the mom told to change from exclusive breastfeeding to formula feeds?	Yes	No	Unknown / N/A
When was the follow-up scheduled to receive HIV DNA PCR results?	Yes	No	Unknown / N/A
Did the mom go back to the clinic to receive the HIV DNA PCR results?	Yes	No	Unknown / N/A
If HIV DNA PCR positive:			
Was a clinical staging done?	Yes	No	Unknown / N/A
Was a CD4 count done?	Yes	No	Unknown / N/A
Was the baby referred for ART?	Yes	No	Unknown / N/A
When? Referral site?			
Waiting time to get an appointment?			
Date of first clinic visit?			
If mother was still breastfeeding, was she advised to stop breastfeeding?	Yes	No	Unknown / N/A
If HIV DNA PCR negative and still breastfeeding:			
Was the mother advised that the child would need a repeat HIV DNA PCR test?	Yes	No	Unknown / N/A
What was mother told in terms of the timing of the next PCR test?	Yes	No	Unknown / N/A
What advice on weaning/ changing milk feeds was the mother given?	Yes	No	Unknown / N/A

PAST MEDICAL HISTORY

HIV Elisa positive	Date:<br>Date:<br>Date:			
HIV DNA PCR positive	Date:<br>Date:<br>Date:			
Any previous HIV testing done?		Yes	No	Unknown / N/A
Is caregiver aware of the results of the previous tests?		Yes	No	Unknown / N/A
Any previous referrals for specialized HIV services?		Yes	No	Unknown / N/A
Bactrim prophylaxis		Yes	No	Unknown / N/A
Yes, for how long				
Previous admissions				
Admission date:				
Reason:				
Risk factors for HIV identified:		Yes	No	Unknown / N/A
Counseled and VCT offered:		Yes	No	Unknown / N/A
Admission date:				
Reason:				
Risk factors for HIV identified:		Yes	No	Unknown / N/A
Counseled and VCT offered:		Yes	No	Unknown / N/A
Admission date:				
Reason:				
Risk factors for HIV identified:		Yes	No	Unknown / N/A
Counseled and VCT offered:		Yes	No	Unknown / N/A

Tuberculosis				
Any symptoms				
Type:	Pulmonary TB	Extra pulmonary TB	Site	
Investigations:	Tuberculin skin test	Nasogastric aspirates	CXR	
Treatment	Duration:	Compliance:	Good	Poor
Patient ever received treatment at traditional healer?				

SOCIAL HISTORY

Is the mother the primary caregiver of the child?	Yes	No	Unknown / N/A
Has the child had different primary caregivers over time?	Yes	No	Unknown / N/A
What was the reason for the change in the primary caregiver?			

Residential area:			Type of shelter:		
Rooms:			Amount of occupant:		
Electricity	YES	NO	Water	YES	NO
Tap inside		Tap outside		None	
Total income of household per month					

Source of income		
Distance to closest clinic	Hospital	Kalafong ART clinic
Transport used	Average cost	
Care dependency grant Received	Amount of grants	

FAMILY HISTORY

	Age	Alive	Healthy	HIV	CD4	ART?	TB Rx?
FATHER							
MOTHER							
CAREGIVER							
SIBLING 1							
SIBLING 2							
SIBLING 3							
SIBLING 4							
SIBLING 5							
OTHER:							

Comments:
____
____
____
____
____
____
____

Medical record review

1. CHILD MEDICAL RECORDS

CLINICAL PARAMETERS

Weight:				% of expected
Z-score:				
Normal for age	Underweight for age	Kwashiorkor	Marasmus	Marasmic kwashiorkor
Length		Head circumference		
Current reason for seeking medical help:				
Current medical problem list:				
WHO clinical staging:				

**STAGE 1:**	
Asymptomatic	Persistent generalized lympadenopathy

**STAGE 2:**		
Hepatosplenomegaly	Papular pruritic eruptions	Seborrhoeic dermatitis
Extensive human papilloma virus infection	Extensive molluscum contagiosum	Fungal nail infections
Recurrent oral ulcerations	Lineal gingival erythema	Angular cheilitis
Parotid enlargement	Herpes zoster	Recurrent / chronic respiratory infections (otitis media, otorrhoea, sinusitis)
**STAGE 3:**		
Moderate unexplained malnutrition not adequately responding to standard therapy (between the 3rd percentile and 60% of expected weight)		
Unexplained persistent diarrhea > 14 days	Unexplained persistent fever > 1 month	
Oral candidiasis	Oral hairy leucoplakia	Acute necrotizing ulcerative gingivitis/periodontitis
Pulmonary TB	Severe recurrent presumed bacterial pneumonia	Chronic HIV associated lung disease including bronchiectasis
Lymphoid interstitial pneumonitis	Unexplained anemia (<8g/dl) and/or neutropenia (<500/mm3) and/or thrombocytopenia (<50000/mm^3) for more than one month.	

STAGE 4:		
Unexplained severe wasting or severe malnutrition not responding to standard treatment		
Pneumocystis jeroveci pneumonia	Recurrent severe presumed bacterial infections e.g.:<br>• Empyema<br>• Pyomyositis<br>• Bone or joint infection<br>• Meningitis	Chronic herpes simplex infection > 1 month
Extra pulmonary TB	Kaposi sarcoma	Esophageal candidiasis
CNS toxoplasmosis (outside neonatal period)	HIV encephalopathy	Cytomegalovirusinfection
Extra pulmonary cryptococcosis including meningitis	Disseminated endemic mycosis	Cryptosporidosis
Disseminated non-tuberculous mycobacterium infection	Candida of trachea, bronchi or lungs	Visceral herpes simplex infection
Acquired HIV associated rectal fistula	Cerebral or B-cell non-Hodgkin lymphoma	Progressive multifocal leukoencephalopathy
HIV associated cardiomyopathy	HIV associated nephropathy	

LABORATORY INVESTIGATIONS

CD4	Percentage:	Absolute:
	HIV viral load	
Full blood count:	Hemoglobin	
	MCV	
	MCH	
	Lymphocyte count	
Albumin	Globulin	

ROAD TO HEALTH CARD

Available	**Yes**	**No**	**Unknown / N/A**
Reason if not available:			
**Immunizations**			
Up to date?	**Yes**	**No**	**Unknown / N/A**
If not what is missed?			
What is the next immunization?			
Is next appointment or immunization date recorded on the chart?	**Yes**	**No**	**Unknown / N/A**
Risk factors for HIV noted on road to Health Card:			
PMTCT code: Interpretation:			
Was coding likely to have been correct?	**Yes**	**No**	**Unknown / N/A**
HIV PCR results	**Yes**	**No**	**Unknown / N/A**
Bactrim prophylaxis	**Yes**	**No**	**Unknown / N/A**
Baby feeding choice noted	**Yes**	**No**	**Unknown / N/A**
Features of HIV infection:	**Yes**	**No**	**Unknown / N/A**
Pneumonia	**Yes**	**No**	**Unknown / N/A**
Persistent diarrhea	**Yes**	**No**	**Unknown / N/A**
Ear discharge	**Yes**	**No**	**Unknown / N/A**
Low weight for age	**Yes**	**No**	**Unknown / N/A**
Unsatisfactory weight gain	**Yes**	**No**	**Unknown / N/A**

Oral thrush	**Yes**	**No**	**Unknown / N/A**
Parotid enlargement	**Yes**	**No**	**Unknown / N/A**
Enlarged lymph glands in one or more of following sites?<br>• Neck<br>• Axilla<br>• Groin	**Yes**	**No**	**Unknown / N/A**
Any other notes written on card regarding HIV:			

2. MATERNAL MEDICAL RECORDS

Medical records:	Available	Not available	Incomplete
Age	G	P	M
Reasons for miscarriage:			
RPR	Partially Rx	Fully Rx	Date:
Infections during pregnancy?<br>1.<br>2.<br>3.		Date:<br>Date:<br>Date:	
Intrapartum procedures<br>1.<br>2.<br>3.		Date:<br>Date:<br>Date:	
VCT	NONE	OFFERED	REFUSED
HIV ELISA	Date:	HIV ELISA	Date:
CD4 count:	Date:	HIV VL	Date:

Referred for antiretroviral therapy?	**Yes**	**No**	**Unknown/NA**
Duration between HIV-test, CD4 count result and referral for ART:			
Antiretroviral therapy:	**Yes**	**No**	**Unknown/NA**

Gestation:	AZT:	HAART:
Duration between referral for ART and appointment at ART clinic:		

Place of birth:	Home	Clinic	Hospital	Unknown

Where the health care providers (doctors, nurses) assisting in the delivery aware of the mothers HIV status?	**Yes**	**No**	**Unknown/not applicable**

Mode of delivery:	**Normal vaginal**	**Caesarean section in labor due to obstetrical indication**	**Elective caesarean section**	**Forceps**	**Vacuum delivery**

Indication for assisted delivery?			
Premature rupture of membranes	**Yes**	**No**	**Unknown/NA**

If yes:	**Spontaneous**	**Induced**	**< 4 hours**	**> 4 hours**

**ARV's in labor**	**No treatment**	**Nevirapine alone**	**Timing of NVP**	**Nevirapine and AZT**	HAART	**Incomplete**

Birth complications:

Feeding counseling:	**None**	**Doctor**	**Sister**	**Other:**
Feeding choice noted	Yes	No	Feeding choice:	

	Not noted	Acceptable	Feasible	Affordable	Sustainable	Safe
